# Preliminary Investigation of Biogenic Amines in Type I Sourdoughs Produced at Home and Bakery Level

**DOI:** 10.3390/toxins14050293

**Published:** 2022-04-20

**Authors:** Giuseppe Mannino, Fortunato Cirlincione, Raimondo Gaglio, Elena Franciosi, Nicola Francesca, Giancarlo Moschetti, Alberto Asteggiano, Claudio Medana, Carla Gentile, Luca Settanni

**Affiliations:** 1 Department of Life Science and Systems Biology, University of Turin, Via Accademia Albertina, 13, 10123 Torino, Italy; giuseppe.mannino@unito.it; 2 Department of Agricultural, Food and Forest Sciences, University of Palermo, Viale delle Scienze, Ed. 5, 90128 Palermo, Italy; fortunato.cirlincione@unipa.it (F.C.); raimondo.gaglio@unipa.it (R.G.); nicola.francesca@unipa.it (N.F.); giancarlo.moschetti@unipa.it (G.M.); 3 Research and Innovation Centre, Edmund Mach Foundation (FEM), Via E. Mach 1, 38098 San Michele all’Adige, Italy; elena.franciosi@fmach.it; 4 Department of Molecular Biotechnology and Health Sciences, University of Palermo, Via Giuria, 5, 10125 Torino, Italy; alberto.asteggiano@unito.it (A.A.); claudio.medana@unito.it (C.M.); 5 Department of Biological, Chemical and Pharmaceutical Sciences and Technologies, University of Palermo, Viale delle Scienze 16, 90128 Palermo, Italy

**Keywords:** biogenic amines, food safety, UPHLC-MS/MS, lactic acid bacteria, MiSeq Illumina, sourdough

## Abstract

During a survey for isolating sourdough lactic acid bacteria (LAB), 20 dough samples produced at the bakery level (BL) or home-made (HM) were collected. An enzyme-based colorimetric method revealed a total biogenic amines (BAs) concentration in the range 41.4–251.8 ppm for six (three BL and three HM) sourdoughs characterised by unpleasant odours. Eight BAs generally investigated in foods were identified and quantified from these six samples by ultra-high performance liquid chromatography tandem mass spectrometry (UHPLC–MS/MS). Only one HM sample contained almost all analysed BAs. Tryptamine was exclusively detected in HM sourdoughs (0.71–24.1 ppm). Putrescine, tryptamine, spermidine, and spermine were the only BAs detected in BL sourdoughs. MiSeq Illumina analysis was applied to study the total bacterial community of sourdoughs. LAB accounted from 67.89 to 92.17% of total bacterial diversity, and *Levilactobacillus brevis* was identified in all six sourdoughs. *Leuconostoc*, *Pediococcus*, and *Weissella* were also dominant. Plate counts detected neither the presence of *Pseudomonas* nor members of the Enterobacteriaceae family, and LAB levels were, on average, barely 5.89 Log CFU/g for BL, and 7.33 Log CFU/g for HM sourdoughs. Data suggested that the microorganisms mainly imputable of BAs formation in sourdough are members of the LAB community.

## 1. Introduction

One of the pathways of amino acid catabolism leads to their decarboxylation in the corresponding amines, namely biogenic amines (BAs), in a process catalysed by decarboxylases widely distributed in living organisms. In particular, bacteria are generally very rich in these enzymatic activities [1].

Some BAs are involved in important and various physiological processes in humans, including brain activity, immune response, and cell growth and differentiation. For example, histamine, produced by histidine decarboxylation, is a powerful vasodilator that also stimulates gastric acid secretion; tyramine, produced by the decarboxylation of tyrosine, has a hypertensive action; serotonin, dopamine, and γ-aminobutyric acid (GABA), produced by decarboxylation of 5-hydroxytryptophan, 3,4-dihydroxyphenylalanine, and glutamic acid, respectively, are important neurotransmitters. On the other hand, other BAs, such as the diamines cadaverine and putrescine, produced by the decarboxylation of lysine and ornithine, respectively, are instead very toxic and responsible for the toxicosis caused by intestinal bacterial alterations [2,3].

Despite the physiological role of some BAs, the ingestion of foods containing high levels of these nitrogen compounds caused by an excessive microbial decarboxylation, determines several adverse effects on consumers’ health. Indeed, though a low amount of exogenous BAs can be rapidly detoxified by serum monoamine oxidase (MAO; EC 1.4.3.4) and diamine oxidase (DAO; EC 1.4.3.22) [4], larger quantities saturate the physiological detoxifying capacities of these enzymes. Toxicological adverse effects include hypertension, smooth muscle contractions, nausea and diarrhoea, heart palpitations, and changes in perception [5,6,7]. Furthermore, BAs can react with nitrites forming the potential carcinogenic nitrosamines [8]. Consequently, BAs have been proposed as food safety indicators [9,10].

It is noteworthy that, though BAs are not necessarily the casual agents of malodours in foods, their concentrations are well correlated with the development of spoilage off-flavours in some products [11,12].

Lactic acid bacteria (LAB) are primarily involved in the fermentation process of several alimentary raw materials [13], but they can also decarboxylate amino acids actively [14]. Thus, BAs are especially detected in fermented foods and beverages [15,16].

On the other hand, in comparison with other fermented foods, sourdoughs and their derived products show low food safety risks [17]. However, del Rio et al. [18] recently reported *Furfurilactobacillus rossiae* (formerly *Lactobacillus rossiae*), typically associated with Type I sourdoughs [19,20], as a producer of putrescine, which is one of the most common BAs in foods, together with histamine and tyramine [21].

Sourdough is a complex microbial environment originated from a mixture of flour and water where LAB and yeasts grow together, affecting the process of mass acidification (mainly LAB) and the increase of dough volume (basically, yeasts) [22]. Sourdough is classified into three types, with Type I refreshed daily using a part of the previous fermentation and considered a traditional process, Type II propagated at the industrial level with inoculated selected strains adapted at high temperatures, and Type III subjected to drying and used at the industrial level to reduce the variability of productions [23].

Even though sourdough fermented products undergo the baking process and fermenting cells are killed, BAs are quite resistant to heating, as confirmed by Kurt and Zorba [24], who estimated that until 90 °C, the effect of heat treatment on BAs is not significant. Consequently, BAs produced during sourdough fermentation can represent a risk in bread and baked goods. However, this topic has been so far underrated.

Here, with the aim of paying major attention to this issue, Type I sourdoughs produced at the bakery as well as house level, and characterised by undesirable odour profiles, were analysed for their BAs content, and the microbial communities were investigated by a culture-independent approach followed by plate counts.

## 2. Results and Discussion

### 2.1. Determination, Identification, and Quantification of Biogenic Amines in Sourdoughs

Scientific evidence suggests that BAs other than histamine should be considered for human health hazards, and the Food and Drugs Administration Agency (FDA) also proposed to use other BAs to evaluate fish freshness [25]. However, except for specific legislation that exclusively regulates the content of histamine in fishery products (European Commission Regulation [26], no similar criteria have been established for other BAs or food products [27]. Concerning fishery products, the European Community established that fish may be considered as decomposed when the histamine level reaches 50 ppm [28]. Anyway, BA levels above 1000 ppm in foods are associated with toxicity phenomena. On the other hand, 100 ppm histamine, or a total of BAs up to 200 ppm are considered acceptable [29].

In this study, 10 dough samples from bakeries and 10 from local artisan producers were collected from throughout the Sicily region (southern Italy), and analysed for the total amounts of BAs by a modified enzyme-based colorimetric method [28]. Only six sourdoughs showed detectable amounts (limit of detection (LOD): 1.35 ppm; limit of quantification (LOQ): 4 ppm) of BAs, whereas 14 samples were characterised by BAs concentrations below the (LOD). The content of BAs of the sourdoughs, the object of investigation, are shown in Table 1. All six sourdoughs positive for BAs detection were also characterised by unpleasant odours, and further processed.

The content of BAs among the HM sourdoughs was higher in the BL ones, with average levels equal to 146.35 and 49.14 ppm, respectively. Moreover, whereas in HM sourdoughs, the variability between samples was considerable, limited variations were observed among BL sourdoughs. Although the three sourdoughs produced at the bakery level had a BA content around 50 ppm, among HM samples, 1HM sourdough was characterised by a BA content above 200 ppm.

Based on these preliminary results, the quantitative and qualitative profile of the eight BAs (histamine, putrescine, cadaverine, 2-phenylethylamine, tyramine, tryptamine, spermidine, and spermine) generally investigated in foods [30] was determined by UHPLC-MS/MS techniques only for the six samples showing detectable BA levels in the enzyme-based colorimetric assay (Table 2).

According to the results from the colorimetric assay, HM samples showed the highest content of BAs with values that were from three- to almost seven-fold higher than those detected in BL samples. Once again, 1HM sourdough contained the highest amount of BAs among HM samples.

Regarding the qualitative amine profile of the investigated sourdoughs, sample 1HM was the only sample containing almost all studied BAs, except for cadaverine, which was not detected in any of the analysed samples. Moreover, 1HM was also the only sample to contain histamine and 2-phenylethylamine. In particular, the detected amount of histamine was about 4% of the total BAs content of 1HM sourdough. Tyramine and putrescine were found at the highest concentrations, representing over 80% of the total BAS content in 1HM and 3HM samples. On the contrary, a higher homogeneous distribution of putrescine, tyramine, spermidine, and spermine was observed for 2HM sourdough. Tryptamine was exclusively detected in HM sourdoughs, ranging between 0.711 and 24.023 ppm. 

Regarding BL sourdoughs, the average value of the sum of the eight BAs was around 25 ppm. For these samples, a reduced BAs variability indicated, as expected, that the preparation conditions of the sourdoughs from bakery were much less variable than those encountered at the home level. Putrescine, tryptamine, spermidine, and spermine were the only BAs detected in BL samples. In particular, putrescine and tryptamine accounted for over 75% of the total BAs content in the three samples. Tyramine content, which exceeded 60% of the total BAs in 1HM and 3HM, represented barely 2% of the total BAs content of BL sourdoughs. Among the different BAs, acute toxic effects are mainly caused by histamine and tyramine. Histamine intoxication causes a symptomatology known as “scombroid fish poisoning” with redness and skin rashes, burning in the mouth, severe headaches, heart palpitations, asthma attacks, and abdominal cramps [31]. Tyramine, a strong vasoconstrictor, causes an intoxication known as “cheese reaction”. This symptomatology is characterised by increased cardiac output, nausea, vomiting, respiratory disorders, increased blood pressure, heart failure, brain hemorrhage, and gastrointestinal disorders also caused by increased enteropathogenic bacteria adhesion to gut mucosa [32]. Among other BAs, putrescine, cadaverine, spermine, and spermidine acute intoxication are less known. Putrescine and cadaverine increase adverse effects of BA intoxication by the inhibition of MAO and DAO [33], enzymes involved in BA catabolism. Spermine and spermidine increase blood pressure and induce respiratory symptoms and neurotoxicity at very high concentrations [34]. Finally, the health risks associated with the intake of tryptamine and 2-phenylethylamine in foods have been insufficiently characterised.

### 2.2. Taxonomic Distribution of Bacterial OTUs

**Figure 1:**
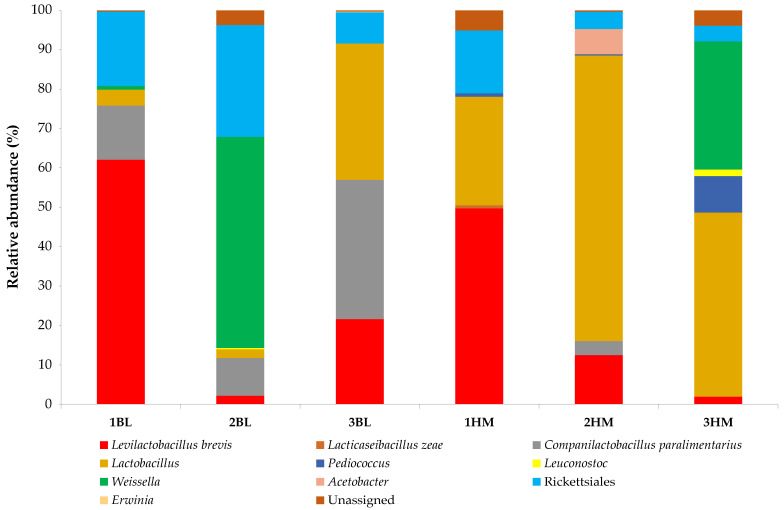
Relative abundances (%) of bacterial taxa identified by MiSeq Illumina in sourdough samples. Abbreviations: BL, bakery-level sourdough; HM, home-made sourdough.

The DNA extracted from the six sourdoughs characterised by a detectable level of BAs was always successfully amplified in the bacterial V3–V4 16S rRNA gene region, and 441,902 paired-end sequences were obtained. Miseq Illumina analysis resulted in a very narrow taxonomy classification, since only lactobacilli, *Leuconostoc*, *Pediococcus*, *Weissella*, *Erwinia*, *Acetobacter*, and Rickettsiales were identified. It has to be noticed that besides *Lactobacillus* genus, the group of lactobacilli includes also the genera *Companilactobacillus*, *Lacticaseibacillus* and *Levilactobacillus*, recently reclassified by Zheng et al. [35]). A small number of taxa have also been identified by the same technology in other traditional fermented products from soybeans [36] and cowpeas [37]. The relative abundances (%) of the operational taxonomy units (OTUs) identified from sourdough samples are shown in Figure 1, where only the OTUs with an individual relative abundance above 0.1%, fixed as a threshold for abundant communities [38], were considered.

LAB dominated all six sourdough samples with unpleasant odours. This bacterial group ranged from 67.89 (in sample 2BL) to 92.17% (in sample 3HM) of the OTUs identified. On average, bakery-level sourdoughs hosted LAB at a lower percentage (80.11%) than that revealed for home-made sourdoughs (86.67%). However, the LAB species composition was quite different among samples. *Lactobacillus* constituted barely 2.16% of total bacterial diversity of sample 2BL, but this genus accounted for 72.60% in sample 2HM, and was present in all sourdoughs analysed. *Lactobacillus* represents the main genus in Type I sourdoughs [22]. Thus, the results registered for the sample 2BL are particularly low. Some lactobacilli were even identified at the species level: *Levilactobacillus brevis* was identified from all six sourdoughs, and represented almost the half of total bacterial diversity (49.70%) in sample 1HM, and even more than half (62.09%) in sample 1BL; *Companilactobacillus paralimentarius* (3.47–35.38%) was detected in four samples, including all three bakery level sourdoughs; *Lacticaseibacillus zeae* was only hosted in sample 1HM at a very low relative abundance (0.81%). Lactobacilli are the typical LAB associated with sourdoughs [39] with *Lvb. brevis* (formerly *Lactobacillus brevis*) and *C. paralimentarius* (formerly *Lactobacillus paralimentarius*), as the main species representative of the obligate heterofermentative and facultative heterofermentative groups, respectively, identified from Italian Type I sourdoughs [40,41,42]. *Lcb. zeae* (formerly *Lactobacillus zeae*) is less common in sourdough, but easily revealed when culture-independent methods are applied to this matrix [43]. The LAB community of both the bakery-level and home-made sourdoughs was also composed of *Leuconostoc*, *Pediococcus*, and *Weissella*. Even though these genera mainly originate from raw materials, and both durum semolina and tender flour are used for bread-making in south Italy [44,45], they showed very interesting properties during sourdough propagation [46], and are able to perform industrial fermentations, in particular *Leuconostoc* and *Weissella* species [47,48]. However, these three genera were found in a few samples, and, basically, at low relative abundance, but it is worth noting that *Weissella* represented 32.55 and 53.63% of the total bacterial community of the samples 3HM and 2BL, respectively, highlighting the important role of Weissellas in sourdough fermentation.

Regarding non-LAB OTUs, Rickettsiales were found in all samples (3.97–28.44%). Generally, their presence is imputable to environmental contaminations [49]; as, being associated to vertebrate and invertebrate hosts [50], they might be hosted in unprocessed raw materials used to propagate sourdoughs. *Acetobacter* (6.43%) were only detected in sample 2HM. However, the presence of *Acetobacter* spp. among sourdough microbial communities has been reported by several authors [51,52,53]. A very low relative abundance (0.14%) was attributed to the genus *Erwinia* in sample 3BL. Also, the presence of this genus might be imputable to raw materials, since *Erwinia* species are reported as growth promoters for wheat [54].

### 2.3. Acidity of Sourdoughs

The level of acidification of the six sourdough samples processed was estimated through pH measurement and TTA (Table 3).

The pH values of the samples collected from bakeries were (on average, 4.59) quite higher than those (on average, 3.78) of the sourdoughs produced at the home level. The last samples showed a pH comparable to those of sourdough produced in Sicily both at the artisanal as well as industrial level [42,47,48]. TTA was negatively and linearly correlated with pH: a low pH corresponded to a high level of TTA. Thus, all bakery sourdoughs were characterised by lower TTA levels than home-made sourdoughs. In particular, TTA levels estimated for HM samples were in the same range of those developed by LAB [46].

### 2.4. Levels of Viable LAB and Yeasts

The microbiological investigations were exclusively performed for the six sourdoughs showing detectable BA levels. Cell densities of TMC (Table 4) of sourdoughs propagated at the bakery level were, on average (5.89 Log CFU/g), consistently lower than those observed for the home-made sourdoughs (7.33 Log CFU/g).

Although TMC levels detected for HM samples were lower than those generally reported for mature sourdoughs, which overcome 8.0 Log CFU/g [55], cell densities registered for BL samples were unusually low for wheat sourdoughs, indicating serious issues related to the microbiological communities of these samples. 

LAB populations of HM sourdoughs (Table 4), similarly to TMC, were around 10^7^ CFU/g. Moreover, in this case, their levels were lower than those commonly registered for mature sourdoughs [56,57]. Among HM samples, however, 2HM was characterised by the lowest TMC and LAB levels, all below 10^7^ CFU/g. Furthermore, mesophilic cocci counted on M17 were below the detection level. No big differences were observed among the four LAB groups (mesophilic rods (on mMRS) and cocci (on M17); typical sourdough LAB (on SDB) and presumptive *Fructilactibacillus sanfranciscensis* (on SFM)) for the samples 1HM and 3HM.

As expected, the levels of LAB found for BL sourdoughs were particularly low. Except the 2BL sample, for which all four LAB groups were in the range 6.23–6.83 Log CFU/g, 1BL showed the presence of only two LAB groups at low levels (4.86 and 5.80 Log CFU/g on mMRS and SDB, respectively), whereas 3BL was characterised by the absence of mesophilic cocci only, and the levels of the other LAB groups were very low (4.09–4.29 Log CFU/g). The absence of bacterial growth on M17 is imputable to the pH of this medium (>6.0), which is too high for the growth of typical sourdough LAB [58]. However, the very low levels of LAB confirmed serious concerns for the fermenting populations of these sourdough samples, because at the end of fermentation, LAB populations in Sicilian Type I sourdoughs account for higher levels [42,44].

Due to the undesirable odours emitted by the sourdoughs, all samples were investigated for the presence of members of the Enterobacteriaceae family, and pseudomonads that are generally involved in the process of generation of unpleasant off-odours [59]. Neither of these two groups were detected in any sample.

Yeasts were also an object of enumeration (Table 4). Only three samples (2BL, 1HM, and 2HM) showed their growth in the range 5.09–5.49 Log CFU/g. Generally, the levels of yeasts in sourdoughs are around two orders of magnitude lower than those of LAB [55,60]. In this work, yeast and LAB numbers were at a ratio 1:100 for 1HM, and 1:10 for 2HM and 2BL.

In all sourdough samples analysed, the levels of LAB were almost superimposable to TMC, clearly showing that the former group dominated the microbial communities.

### 2.5. Multivariate Analysis

**Figure 2:**
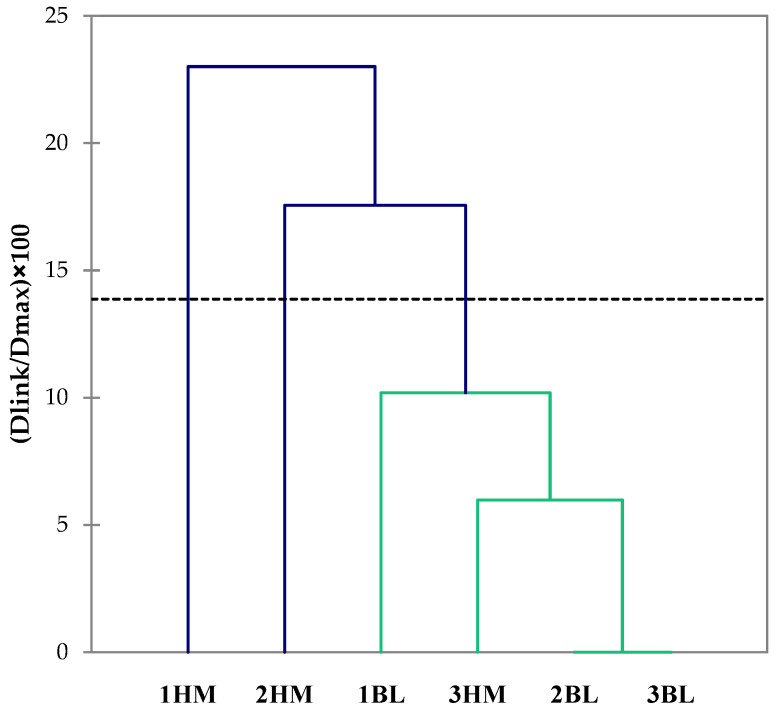
Dendrograms resulting from HCA based on values of microbiological characteristics and concentration of biogenic amines. The dissimilarity among sourdoughs was measured by Euclidean distance, whereas cluster aggregation was achieved by single linkage. Abbreviations: BL, bakery-level sourdough; HM, home-made sourdough.

HCA classified the sourdoughs with unpleasant odours in accordance with their mutual dissimilarity and relationship (Figure 2) by using a total of 16 variables, including microbiological characteristics and the concentrations of biogenic amines of sourdoughs.

HCA is a hierarchical ascendant analysis where the samples examined (sourdoughs) are graphically represented by a dendrogram from the closest one, i.e., the most similar, to the furthest apart, which is the most different sample [61]. Considering a dissimilarity level of 14% provided by the program STATISTICA, 1BL, 2BL, 3BL, and 3HM sourdoughs formed a single cluster and they were clearly separated from 1HM and 2HM sourdoughs. In particular, the dissimilarity found for these two sourdoughs was imputable to their putrescine, cadaverine, and spermidine content.

## 3. Conclusions

Among the 20 sourdoughs of this survey, six samples, all characterized by unpleasant odours, showed detectable levels of BAs. The microbiological analyses conducted by classical plate counts detected neither the presence of *Pseudomonas* nor members of the Enterobacteriaceae family, which are generally involved in production of putrid and sulphuric odours. The levels of LAB were quite lower for the samples collected from bakeries, but Illumina analysis showed that the majority of OTUs were ascribed to LAB genera and species commonly associated to mature sourdoughs. Only *Erwinia* was identified among the Enterobacteriaceae family. However, all sourdoughs were positive for the presence of BAs, with those produced at the home level showing the highest concentrations. So far, BAs have been reported as spoilage off-flavours in fish and meat products, and this study provides evidence that sourdoughs should be better investigated for these aspects.

## 4. Materials and Methods

### 4.1. Sourdough Collection

Ten home-made (HM) sourdoughs and ten sourdoughs produced at the bakery level (BL) were collected throughout the Sicily region (southern Italy) (Table 5). The sampling plan included HM sourdoughs because breads produced at the home level are consumed daily in southern Italy [62], and to better characterise these artisanal leavening agents for their BAs content for the first time.

All BL sourdoughs were propagated through mechanical fermenters applying the conditions reported in Table 5, and the mixing was carried out at the minimum speed. HM sourdoughs were subjected to the classical refreshment by manual kneading, and the propagation conditions are reported in Table 5. All producers excluded, at any extent, the addition of commercial baker’s yeast. In order to pick up sourdoughs at the highest acidity level, the analysed samples were collected in duplicate just before daily refreshment. Sourdoughs (almost 100 g) were transferred into 200 mL volume sterile cups (Anicrin, Scorzé, Italy) and transported under refrigeration in thermally-insulated boxes to the laboratory of Agricultural Microbiology of Palermo University (Italy), where they were immediately analysed. Each sourdough was sampled after two weeks to obtain two independent replicates per producer.

### 4.2. Screening for Biogenic Amines

The presence of BAs in all 20 sourdoughs was determined by a modified enzyme-based colorimetric method according to Yeh, Lin, and Hwang [28], with minor changes. Samples were preliminary prepared by protein precipitation using trichloroacetic acid. Briefly, 20 mL of 20% (*v*/*v*) trichloroacetic acid was added to 5 g of the sample and homogenized for 10 min. Then, 10 mL of homogenate was diluted up to 100 mL using distilled water. After a clean-up step via centrifugation (10 min at 5000× *g*, 4 °C) and filtration, the supernatant was collected, and the pH was adjusted to 9 using 1 M KOH. Colour developing reagent was prepared mixing 1.5 M Tris buffer (pH 9.0), 400 mM 4-aminoantipyrine, and 40 mM phenol in a ratio 4:1:1 (*v*/*v*/*v*), respectively. Reaction mixture was prepared mixing 1 mL of each sample, 450 µL colour developing reagent, 500 µL of 300 mU/mL diamine oxidase, 50 µL of 175 U/mL horseradish peroxidase, and water up to 25 mL. After stirring and incubation at 50 °C in a water bath for 1 h, the absorbance of the reaction mixture was measured at 505 nm against a blank: a reaction mixture containing distilled water instead of the sample. 

Quantification was performed using an external calibration curve of histamine dihydrochloride in the range of 1–80 ppm. The total amount of biogenic amines in sourdough samples was then expressed as ppm of histamine equivalents (HE) per grams of fresh weight using the following equation:

HE = C × V_1_ × V_2_/V_3_ × W (1)

where C is the HE concentrations in the reaction mixture of the assay and expressed as mg/mL; V1 is the final volume expressed in mL to which the aliquots have been first diluted (in our experimental conditions, 100); V2 is the reaction volume expressed in mL and used for the detection and quantification of BAs in sample extracts (in our experimental conditions, 25); V3 is the volume expressed in mL and taken from each sample extract and diluted up to 100 mL (in our experimental condition, 10); W is the weight of the sourdoughs expressed in g and employed for the extraction process. LOD and LOQ were calculated as previously described [63], according to Equation (2) and Equation (3), respectively:

LOD = 3.3 × σ/S(2)

LOQ = 10 × σ/S(3)

where σ is the standard deviation of y-intercepts on *x*-axis of the calibration curve, whereas S is the slope of the regression curve.

### 4.3. UHPLC-MS/MS Evaluation of Sourdough Biogenic Amines

BAs were extracted only from the sourdough samples that showed detectable levels of BAs during the preliminary screening using 0.1% (*v*/*v*) HCl and 1:5 (*w*/*v*) as the solvent ratio. Extraction was monitored by adding 50 ng of 2-PEA (phenylethylamine) to the solvent as an extraction standard. The solution containing the sample was vortexed for 2 min, sonic-bathed for 10 min, and, in order to separate solid from liquid components, centrifuged at 12,000× *g* for 20 min at 4 °C. In order to perform an exhaustive extraction, the process was repeated twice, and the aqueous layers combined. The resulting solution was diluted 1:10 (*v*/*v*) in 5 mM HFBA (heptafluorobutanoic acid) for the UHPLC run. HFBA was used as an ion pairing agent for the highly polar BAs. The analytical system chosen was a Shimadzu LCMS 8045 (Shimadzu Milan, Italy) equipped with a NexeraXR UHPLC pump module (Shimadzu Milan, Italy). Elution was performed at 0.3 mL/min by a solvent gradient of A (5 mM HFBA in H_2_O) and B (5 mM HFBA in MeOH) on a Luna C18(2) reversed phase column (150 mm × 3 mm; particle size, 3 µm; pore size, 100 Å) (Phenomenex, Bologna, Italy). The gradient was set as follows: in the first 2 min of the run, it was kept in isocratic conditions (20% B), then it reached 50% of B in 1 min; from 3 to 10 min, the gradient was readjusted to reach 100% of B and 100% B was kept for 5 min; finally, the column was allowed to recondition for 6 min.

Regarding the mass spectrometer, source parameters were set as follows: nebulising gas flow: 3 L min^−1^, desolvation line temperature: 250 °C, heat block temperature: 400 °C, drying gas flow: 10 L/min.

The mass spectrometer operated in MRM positive ion mode, monitoring the transitions of 112.0 > 95.1 (histamine), 103.1 > 86.2 (cadaverine), 89.2 > 72.15 (putrescin), 122.1 > 105.1 (2-PEA), 138.2 >121.2 (tyramine), 161.1 > 144.1 (triptamine), 146.3 > 72.1 (spermidine), 203.3 >112.1 (spermine). All standards used were purchased from Merck (Darmstadt, Germany).

### 4.4. Culture-Independent Microbiological Analysis

#### 4.4.1. DNA Extraction

Total DNA from each sourdough was extracted using the Power Food™ Microbial DNA Isolation Kit (Mo Bio Laboratories Inc., Carlsbad, CA, USA) following the manufacturer’s instructions. The resulting DNA was then purified with the PowerClean DNA Cleanup Kit (Mo Bio Laboratories Inc., Carlsbad, CA, USA). DNA was quantified by Nanodrop8800 Fluorospectrometer (Thermo Scientific, Wilmington, NC, USA). The DNA collected from the sourdoughs positive for the presence of BAs (considering both duplicate samples of the two distinct collection days) was pooled as two individual samples per pool, filling in one pool per sourdough. A proper balance of the DNA quantity was performed in each pool in order to obtain an equal representation for each individual sourdough.

#### 4.4.2. Miseq Library Preparation and Illumina Sequencing

Amplicon library preparation, quality and quantification of pooled libraries, and pair-end sequencing using the Illumina MiSeq system (Illumina, San Diego, CA, USA) were performed at the Sequencing Platform, Fondazione Edmund Mach (FEM, San Michele all’Adige, Italy). Briefly, for each sample, a 464-nucleotide sequence of the V3–V4 region [64,65] of the 16S rRNA gene (*Escherichia coli* positions 341 to 805) was amplified. Unique barcodes were attached before the forward primers to facilitate the pooling and subsequent differentiation of samples. To prevent preferential sequencing of the smaller amplicons, the amplicons were cleaned using the Agencourt AMPure kit (Beckman Coulter, Brea, CA, USA) according to the manufacturer’s instructions; subsequently, DNA concentrations of the amplicons were determined using the Quant-iT PicoGreen dsDNA kit (Invitrogen, Waltham, MA, USA) following the manufacturer’s instructions. In order to ensure the absence of primer dimers, and to assay the purity, the generated amplicon libraries quality was evaluated by a Bioanalyzer 2100 (Agilent, Palo Alto, CA, USA) using a High Sensitivity DNA Kit (Agilent). Following the quantitation, cleaned amplicons were mixed and combined in equimolar ratios.

#### 4.4.3. Illumina Data Analysis and Sequences Identification by QIIME2

Raw paired-end FASTQ files were demultiplexed using idemp (https://github.com/yhwu/idemp/blob/master/idemp.cpp, accessed on 19 October 2021), and imported into Quantitative Insights Into Microbial Ecology (Qiime2, version 2018.2: http://library.qiime2.org, accessed on 23 March 2022). Sequences were quality-filtered, trimmed, de-noised, and merged using DADA2 [66]. Chimeric sequences were identified and removed via the consensus method in DADA2. Taxonomic and compositional analyses were carried on by using the feature-classifier plugin (https://github.com/qiime2/q2-feature-classifier, accessed on 19 October 2021). A pre-trained Naive Bayes classifier based on the Greengenes 13_8 99% Operational Taxonomic Units (OTUs) database, which had been previously trimmed to the V4 region of 16S rDNA, bound by the 341F/805R primer pair, was applied to paired-end sequence reads to generate taxonomy tables. Data generated by MiSeq Illumina sequencing were deposited in the NCBI Sequence Read Archive (SRA), and are available under Acc. No. PRJNA772561.

### 4.5. Physicochemical Parameters and Microbiological Analysis

The pH values were measured using the pH meter BASIC 20+ (Crison Instrument S.A., Barcelona, Spain). Total titratable acidity (TTA) was determined using 10 g of each sample with 0.1 N NaOH (expressed in terms of mL of NaOH). All sourdoughs were previously homogenized in 90 mL distilled water with the stomacher BagMixer^®^ 400 (Interscience, Saint Nom, France) for 2 min at maximum speed.

Ten grams of each sourdough were also homogenized in 90 mL of Ringer’s solution and subjected to the decimal serial dilution to perform microbiological analysis. Plate count agar (PCA) was used for total mesophilic count (TMC) after 72 h incubation at 30 °C under aerobiosis. mMRS and M17 were used for the development of rod- and coccus-shaped LAB, respectively, after 48 h incubation at 30 °C under anaerobic conditions by means of a hermetically sealed jar added with the AnaeroGen AN25 system. Sourdough LAB were specifically investigated on sour dough bacteria (SDB) [67] and San Francisco medium (SFM) [68], both incubated for 48 h at 30 °C. Except PCA, all media used for LAB were added with cycloheximide (10 mg/mL) to avoid fungal growth. Members of the Enterobacteriaceae family were grown on Violet Red Bile Glucose Agar (VRBGA), incubated at 37° C for 24 h. Pseudomonads were inoculated on *Pseudomonas* agar base (PAB) added with Cetrimide Fucidin Cephaloridine (CFC) supplement, incubated at 25 °C for 48 h; yeasts were investigated on yeast peptone dextrose (YPD) agar containing chloramphenicol (0.1 mg/mL) to inhibit bacterial growth, incubated at 25 °C for 48 h. All media and chemicals were purchased from Oxoid (Milan, Italy). Plate counts were performed in triplicate on each replicate sample to obtain a total of six data per sourdough.

### 4.6. Statistical Analyses

Microbiological and chemical data were subjected to one-way analysis of variance (ANOVA) using XLStat software version 7.5.2 for Excel (Addinsoft, New York, NY, USA). The Tukey’s test was applied for pairwise comparison. Statistical significance was attributed to *p* < 0.05.

In addition, an explorative multivariate analysis was employed to investigate the relationships among sourdoughs. A hierarchical cluster analysis (HCA) (joining, tree clustering) was carried out for grouping the sourdoughs according to their dissimilarities, measured by Euclidean distances, whereas cluster aggregation was based on Ward’s method [69]. The input matrices used for HCA consisted of microbiological characteristics and the concentrations of biogenic amines of sourdoughs. Statistical data processing and graphic construction were achieved by using STATISTICA software version 10 (StatSoft Inc., Tulsa, OK, USA).

## Figures and Tables

**Table 1:** Total biogenic amine content of sourdoughs.

Sample	Biogenic Amine Content (HE) ppm
1BL	62.647 ± 5.407
2BL	41.176 ± 2.912
3BL	43.600 ± 1.664
4BL	<LOD
5BL	<LOD
6BL	<LOD
7BL	<LOD
8BL	<LOD
9BL	<LOD
10BL	<LOD
1HM	251.764 ± 23.292
2HM	95.882 ± 2.496
3HM	91.390 ± 8.943
4HM	<LOD
5HM	<LOD
6HM	<LOD
7HM	<LOD
8HM	<LOD
9HM	<LOD
10HM	<LOD

Abbreviations: BL, bakery-level sourdoughs; HM, home-made sourdoughs; LOD, limit of detection.

**Table 2:** Concentration (ppm) of biogenic amines in traditional sourdoughs.

Biogenic Amine	Samples						Statistical Significance ^1^
	1BL	2BL	3BL	1HM	2HM	3HM	
Histamine	<LOD ^b^	<LOD ^b^	<LOD ^b^	6.733 ± 0.201 ^a^	<LOD ^b^	<LOD ^b^	***
Putrescine	8.831 ± 0.524 ^c^	14.063 ± 0.584 ^b^	9.001 ± 0.649 ^c^	37.364 ± 1.239 ^a^	15.981 ± 0.923 ^b^	11.702 ± 0.634 ^c^	***
Cadaverine	<LOD^a^	<LOD ^a^	<LOD ^a^	<LOD ^a^	<LOD^a^	<LOD^a^	N.S.
2-Phenylethylamine	<LOD ^b^	<LOD ^b^	<LOD ^b^	0.299 ± 0.012 ^a^	<LOD ^b^	<LOD ^b^	***
Tyramine	0.178 ± 0.004 ^c^	0.307 ± 0.004 ^c^	0.354 ± 0.021 ^c^	112.583 ± 6.377 ^a^	4.634 ± 0.398 ^c^	47.874 ± 3.203 ^b^	***
Triptamine	<LOD ^d^	<LOD ^d^	<LOD ^d^	0.258 ± 0.002 ^c^	24.234 ± 0.318 ^a^	0.711 ± 0.038 ^b^	***
Spermidine	10.205 ± 0.056 ^b^	6.815 ± 0.084 ^c^	9.553 ± 0.366 ^bc^	10.294 ± 0.046 ^b^	20.557 ± 2.649 ^a^	6.587 ± 0.383 ^c^	***
Spermine	6.323 ± 0.09 ^c^	2.666 ± 0.114 ^f^	5.108 ± 0.055 ^d^	8.879 ± 0.142 ^b^	14.287 ± 0.132 ^a^	3.471 ± 0.064 ^e^	***

^1^ Data within a line followed by the same letter are not significantly different according to Tukey’s test. *p* value: *** *p* < 0.001. Abbreviations: BL, bakery-level sourdoughs; HM, home-made sourdoughs; LOD, below the detection limit; N.S., not significant.

**Table 3:** Characteristics of sourdoughs.

Sample	pH	TTA
1BL	4.74 ± 0.01 ^a^	8.80 ± 0.30 ^d^
2BL	4.83 ± 0.01 ^a^	3.92 ± 0.40 ^e^
3BL	4.20 ± 0.01 ^b^	9.65 ± 0.55 ^d^
1HM	3.87± 0.01 ^c^	15.80 ± 0.30 ^b^
2HM	3.78 ± 0.09 ^cd^	27.40 ± 0.40 ^a^
3HM	3.69 ± 0.11 ^d^	14.05 ± 0.65 ^c^
Statistical significance	***	***

Results indicate mean values ± SD of four determinations (carried out in duplicate for two independent samplings). Data within a column followed by the same letter are not significantly different according to Tukey’s test. *p* value: *** *p* < 0.001. Abbreviations: BL, bakery-level sourdoughs; HM, home-made sourdoughs; TTA, total titratable acidity.

**Table 4:** Microbiological characteristics of sourdoughs.

Sample	Microbial Loads							
	PCA	mMRS	M17	SDB	SFM	VRBGA	PAB	YPD
1BL	5.77 ± 0.19 ^c^	4.86 ± 0.13 ^c^	<2 ^c^	5.80 ± 0.15 ^c^	<2 ^e^	<1 ^a^	<2 ^a^	<2 ^c^
2BL	6.86 ± 0.17 ^b^	6.51 ± 0.18 ^b^	6.23 ± 0.10 ^b^	6.83 ± 0.21 ^b^	6.59 ± 0.11 ^c^	<1 ^a^	<2 ^a^	5.33 ± 0.14 ^ab^
3BL	5.06 ± 0.17 ^d^	4.29 ± 0.17 ^d^	<2 ^c^	4.09 ± 0.11 ^d^	4.21 ± 0.13 ^d^	<1 ^a^	<2 ^a^	<2 ^c^
1HM	7.54 ± 0.16 ^a^	7.58 ± 0.12 ^a^	7.35 ± 0.18 ^a^	7.46 ± 0.19 ^a^	7.50 ± 0.10 ^a^	<1 ^a^	<2 ^a^	5.09 ± 0.13 ^b^
2HM	6.69 ± 0.17 ^b^	6.53 ± 0.13 ^b^	<2 ^c^	6.50 ± 0.13 ^b^	6.39 ± 0.15 ^c^	<1 ^a^	<2 ^a^	5.49 ± 0.19 ^a^
3HM	7.76 ± 0.09 ^a^	7.50 ± 0.11 ^a^	7.57 ± 0.18 ^a^	7.28 ± 0.12 ^a^	7.20 ± 0.10 ^b^	<1 ^a^	<2 ^a^	<2 ^c^
Statistical significance	***	***	***	***	***	N.S.	N.S.	***

Units are log CFU/g. Results indicate mean values ± S.D. of six plate counts (carried out in triplicates for two independent samplings). Data within a column followed by the same letter are not significantly different according to Tukey’s test. *p* value: *** *p* < 0.001. Abbreviations: BL, bakery-level sourdoughs; HM, home-made sourdoughs; PCA, plate count agar for total mesophilic count; mMRS, de Man–Rogosa–Sharpe modified agar for mesophilic rod LAB; M17, medium 17 agar for mesophilic coccus LAB; SDB, sourdough bacteria agar for sourdough LAB; SFM, San Francisco medium for sourdough LAB; PAB, *Pseudomonas* agar base for pseudomonads; VRBGA, Violet Red Bile Glucose Agar for members of the Enterobacteriaceae family; YPD, yeast peptone dextrose agar for yeast; N.S., not significant.

**Table 5:** Characteristics of sourdoughs.

Sample	City (Province) ^1^	Final Product	Age ^2^ (Years)	Type of Flour	DY	DY Factors ^3^			Fermentation Temperature	Fermentation Duration (h)
						Sourdough Inoculum (g)	Flour/ Semolina (g)	Water (mL)		
1BL	Palermo (PA)	Bread	1	Tender flour	275	100	100	75	22 °C	15
2BL	Piana degli Albanesi (PA)	Bread	2	Durum semolina	140	100	500	100	ambient	24
3BL	Piana degli Albanesi (PA)	Bread	3	Durum semolina	160	100	1000	500	20 °C	48
4BL	Cinisi (PA)	Pastry	1	Tender flour	246	100	100	46	28	4
5BL	Palermo	Pastry	70	Tender flour	225	100	120	50	28	3.5
6BL	Carini (PA)	Pizza	1	Tender flour	400	100	50	50	ambient	12
7BL	Castronovo (PA)	Bread	6	Durum semolina	250	100	100	50	ambient	8
8BL	Piana degli Albanesi (PA)	Bread	4	Durum semolina	400	100	50	50	22	6
9BL	Castelbuono (PA)	Pizza	50	Tender flour	300	100	100	100	ambient	24
10BL	Piana degli Albanesi (PA)	Bread	60	Durum semolina	185	100	1000	750	22	4
1HM	Catania (CT)	Bread	2	Flour 50%/semolina 50%	300	100	60	20	ambient	24
2HM	Alcamo (TP)	Focaccia pizza	>100	Tender flour	350	100	50	25	ambient	10
3HM	Rocca di Caprileone (ME)	Pizza	>100	Flour 40%/semolina 60%	230	100	135	75	ambient	12
4HM	Piana degli Albanesi (PA)	Bread	1	Tender flour	350	100	50	25	ambient	24
5HM	Modica (RG)	Bread	3	Durum semolina	260	100	100	60	20	12
6HM	Villabate (PA)	Bread	2	Durum semolina	250	100	100	50	ambient	10
7HM	Sciacca (AG)	Bread	40	Durum semolina	250	100	100	50	ambient	12
8HM	Sciacca (AG)	Pizza	60	Durum semolina	250	100	100	50	ambient	18
9HM	Camporeale (PA)	Bread	30	Wholemeal flour	400	100	50	50	8	120
10HM	Naso (ME)	Pastry	>40	Durum semolina	225	100	360	350	ambient	12

^1^ Province codes: CT, Catania; ME, Messina; PA, Palermo; TP, Trapani. ^2^ Years from first propagation. ^3^ The factors of DY formula were reported per 100 g of sourdough inoculum. Abbreviations: DY, dough yield (weight of dough/weight of flour × 100); BL, bakery-level sourdoughs; HM, home-made sourdoughs.

## Data Availability

All data included in this study are available upon request by contacting the corresponding author.
